# The interplay of gender, social context, and long-term unemployment effects on subjective health trajectories

**DOI:** 10.1186/s12889-021-10324-8

**Published:** 2021-02-04

**Authors:** Laura Altweck, Stefanie Hahm, Holger Muehlan, Tobias Gfesser, Christine Ulke, Sven Speerforck, Georg Schomerus, Manfred E. Beutel, Elmar Brähler, Silke Schmidt

**Affiliations:** 1 Department of Health and Prevention, University of Greifswald, Robert-Blum-Str. 13, 17489 Greifswald, Germany; 2 Department of Psychiatry and Psychotherapy, Leipzig University Medical Center, Liebigstraße 18, Haus B, 04103 Leipzig, Germany; 3 Johannes Gutenberg-University, Mainz, Department of Psychosomatic Medicine and Psychotherapy, University Medical Center, Untere Zahlbacher Str. 8, 55131 Mainz, Germany

**Keywords:** Unemployment, Health, Life satisfaction, Social context, Gender, Panel data, Growth modelling

## Abstract

**Background:**

While a strong negative impact of unemployment on health has been established, the present research examined the lesser studied interplay of gender, social context and job loss on health trajectories.

**Methods:**

Data from the German Socio-Economic Panel was used, which provided a representative sample of 6838 participants. Using latent growth modelling the effects of gender, social context (East vs. West Germans), unemployment (none, short-term or long-term), and their interactions were examined on health (single item measures of self-rated health and life satisfaction respectively).

**Results:**

Social context in general significantly predicted the trajectories of self-rated health and life satisfaction. Most notably, data analysis revealed that West German women reported significantly lower baseline values of self-rated health following unemployment and did not recover to the levels of their East German counterparts. Only long-term, not short-term unemployment was related to lower baseline values of self-rated health, whereas, in relation to baseline values of life satisfaction, both types of unemployment had a similar negative effect.

**Conclusions:**

In an economic crisis, individuals who already carry a higher burden, and not only those most directly affected economically, may show the greatest health effects.

**Supplementary Information:**

The online version contains supplementary material available at 10.1186/s12889-021-10324-8.

## Background

For most people, employment is the foundation to a stable life, enabling us to maintain our sense of purpose and shape our daily life. Beyond the apparent financial strain [1], unemployment can lead to a change in a person’s time structure, their social relationships and in their identity [2, 3]. The non-financial costs of unemployment often have a stronger association with well-being during this time [4]. Overall, the negative relationship between unemployment and health is widely reported in the literature [5–10]. However, what if a whole group of society faces unemployment simultaneously, as seen currently during the COVID-19 crisis?

The set-point theory posits that in the short-term subjective well-being fluctuates following major life events and in the long-term values return to a predetermined ‘set point’ [11–13]. In regards to unemployment, an improvement in health is generally seen after reemployment (e.g., [3]). However, Clark et al. [6] examined the long-term effect of job loss and reemployment on life satisfaction and found that some people did not return to their baseline values many years after the end of their unemployment.

The effects of unemployment on different aspects of health are often used interchangeably. Unemployed individuals have reported significantly lower mental health, subjective physical health and marital, family and life satisfaction compared to their employed counterparts [3, 14], but a non-significant relationship with objective physical health [3] or psychiatric caseness [14] is seen. To address these differential effects of job loss, the present study draws on the World Health Organization’s [15] holistic definition of health – i.e. physical, mental, and social well-being as opposed to the mere dichotomy of ill versus healthy – and examines two different aspects of general health status: self-rated health (a one-item proxy for subjective health) and life satisfaction (cognitive aspect of well-being).

### Short- versus long-term unemployment

Another aspect to consider is short-term as opposed to long-term unemployment, where the latter is largely associated with worse health [3, 6, 16–18]. While individuals who had experienced repeated unemployment compared to those who had never been unemployed reported significantly worse mental health and lower life satisfaction [19], individuals who had experienced single unemployment compared to the other groups (i.e., repeated unemployment or no experience at all) only significantly differed in their satisfaction with income and employment [20]. One can infer that short-term unemployment may impact directly on related matters like finances, while a broader impact on life manifests following longer or repeated experience of unemployment.

Long-term unemployment has often been defined as short as six months [21] or one year or more [22]. While the length of unemployment worsens the effects of job loss on health, this effect does not appear to remain constant. Within the first year of unemployment mental health scores continually fluctuate between deterioration and recovery [7], while after a few years an habituation effect is seen and the increase in health risks plateau [23]. These findings suggest that six to 12 months is not sufficient to adjust to unemployment and for its effect to become fully evident. Therefore, in the current study we defined long-term unemployment as being registered unemployed in two consecutive years.

Lastly, McKee-Ryan et al.’ [3] meta-analysis showed that the effect of longer unemployment on subjective physical health was stronger than on life satisfaction. So we propose the following:

- *H1: The effects of a) long-term* versus *short-term unemployment on self-rated health will be more negative and b) the effects of short- and long-term unemployment will be similar on life satisfaction.*

### Social context

Returning to the overarching research interest: how does mass unemployment affect the well-being in a population? Data from previous occurrences of mass unemployment, help draw parallels to similar circumstances: like the global financial crisis in 2009, the European refugee crisis in 2015 or the current COVID-19 crisis [24]. The present study examines unemployment immediately following the German reunification in 1989/1990. This data is particularly useful in the investigation of unemployment, because in this period East Germans were confronted with a major change in economic policies and their employment system. The government of the former socialist East Germany emphasized the importance of the work force and it had been every citizen’s basic right but also their obligation to take up employment [25]. After reunification, most East Germans faced involuntary retraining from the job centre, a change in workplace or unemployment. The 1991 annual unemployment statistic by the German Federal Employment Agency recorded levels of 10.3% in East Germany and so nearly double to the 6.3% in West Germany [26]. In Struck et al.’ [27] study, 33% of East Germans reported a change in their job role shortly after reunification and 69% reported a change in their workplace, while Mayer [28] reported that 40% had experienced job loss by 1995. In contrast, in West Germany a stable rate of unemployment was present and affected the same groups continuously [29].

In sum, following the reunification unemployment presented differently in East versus West Germany: in the former, unemployment was unexpected and forced on a large scale (e.g., through plant closures), while the latter group grew accustomed to unemployment and the low prospects of reemployment. Then, especially in the West German group the reciprocal relationship between unemployment and health should not be highlighted. Analyses with panel data have shown that unemployed individuals already reported lower health levels prior to becoming unemployed [30] and may have become unemployed as a result of their lower health. In the East German sample, it is less likely that the relationship between unemployment and health would have been reciprocal, because first, unemployment did not exist in former East Germany and so was a novel experience, and second, it presented itself on a large scale and was unpredictable.

High regional unemployment has also been found to be associated with significantly more functional somatic symptoms [31], psychological symptoms [32], and lower subjective well-being [33]. Turner [34] for instance found that in communities with medium to high unemployment rates, current unemployment was associated with higher levels of depression and physical illness. However high regional unemployment in general is associated with lower health in both employed and unemployed individuals living in the region [3, 35] and instead it may be the prospect of reemployment that plays a greater role [36]. Thus we propose that:

- *H2: Unemployment will have a stronger impact on health in the East compared to the West German sample.*

### Gender and unemployment

In former East Germany the discrepancy in income and employment rates between men and women was low [37] and women were encouraged and supported to easily combine their roles as mother, wife and worker [38]. In contrast, in West Germany, in the end 1980s, only 50% of women were in employment [39] and instead most were housewives. After reunification, East German women faced grave discrimination in the more traditionally gender-role oriented West German employment system [27] and faced twice as much job insecurity compared to their male counterparts(female: 31.4%, male: 13.1% [18]).

In general, men assign greater importance to work, which can be explained by traditional gender roles: men are the breadwinners of the family and bear the responsibility of providing for them, while women care for the children and run the household. So along these lines it would be ‘easier’ for women to become unemployed, because, first, they would likely not be the main source of income, and, second, they could transition to the role of housewife, instead of a vacuum of ‘no employment’. By comparison, men would experience the effects of no longer fulfilling their traditional roles. Van der Meer [40] found that while men gain most of their status from their job, women attain their status from several sources. Forret et al. [41] further showed that men with children are more likely to see unemployment as a defeat, while women perceive it as an opportunity.

While women’s participation in the labour market even now is significantly lower than men’s [42], traditional gender roles are changing. Strandh et al. [43] purported that the associations between unemployment, gender and psychological distress are dependent on the social context and compared samples from Ireland and Sweden. They found that compared to Irish men, women reported lower distress following unemployment, whereas Swedish, unemployed women reported greater distress than their male counterparts. The authors explained that Sweden has a large female labour force and the society is strongly defamilised, whereas the opposite is the case in Ireland, which follows more traditional gender roles. Hammarström et al. [44] also studied unemployment in a Swedish sample and similarly found no gender effect. As East German women after reunification similarly did not embody traditional gender roles and had a high participation in the labour market, this group is comparable to the Swedish samples. In this vein we propose that:

- *H3: The interaction effect of unemployment having a stronger impact on health in the East* versus *West German sample should be stronger in the female compared to the male sample.*

## Methods

### Sample

Data from the Core-Study of the German Socio-Economic Panel (SOEP) was used. This is an annual representative longitudinal study of private households from 1984 until present [45, 46]. While some core constructs are measured annually (e.g., household structure, employment history, income), others are measured once or at less frequent intervals (e.g., self-rated health). Data from the GSOEP has been used by other research groups to examine the association between unemployment and life satisfaction (e.g., [6, 8]). However, first, the effects on self-rated health and, second, the specific effects of unemployment after German reunification on self-rated health and life satisfaction has not been investigated with this data.

The GSOEP first collected data from families in West Germany and is particularly interesting for the current study as a group of East German participants joined directly after reunification. Participants outside of working age – i.e., 18–65 years – in 1992 and with missing data were excluded (*N*_total_ = 13,397, *N*_18-65years_ = 11,684). As a result, the current analyses were run on a sample of 6838 participants (*N*_East_ = 2493, *N*_West_ = 4345). The demographics in Table 1 show large differences between context and gender. Compared to male West Germans, the East German participants reported a significantly lower household income, were older, more likely to be in a partnership, less likely to have completed middle but more likely to have completed high education, more likely to be in no or part-time employment, and were more likely to be unemployed from 1991 to 1996 (*p* < .05). Female compared to male West Germans were also significantly older and reported a lower household income, but generally showed lower education rates, and more likely to be in no or part-time employment (*p* < .05). The unemployment rates for West German women fluctuated and they were more likely to be unemployed in 1993 and 1994, but less likely in the other years.

**Table 1:** Socio demographics by social context, and gender: Means (Range), SD / Frequencies (Percent)

	East		West	
	Female	Male	Female	Male
Age	40.05 (18–65), SD = 12.77	40.23 (18–65), SD = 12.79	39.13 (18–65), SD = 13.29	38.31 (18–65), SD = 13.40
Household income	1372.20 (179–3835), SD = 564.18	1459.82 (179–3835), SD = 592.58	2056.28 (0–10,737), SD = 1066.66	2158.08 (256–10,737), SD = 1004.38
Partnership Status				
In partnership	1273 (73.25)	1242 (73.02)	1992 (68.06)	1818 (63.06)
Not in partnership	465 (26.75)	459 (26.98)	935 (31.94)	1065 (36.94)
Education				
Low	1234 (69.40)	1100 (64.21)	3356 (84.36)	3129 (78.03)
Middle	66 (3.71)	215 (12.55)	387 (9.73)	441 (11.00)
High	478 (26.88)	398 (23.23)	235 (5.91)	440 (10.97)
Employment type				
Not employed	395 (28.66)	177 (15.39)	1171 (36.29)	324 (11.52)
Full-time	772 (56.02)	1060 (92.17)	1194 (37.00)	2590 (92.07)
Part-time	167 (12.12)	48 (4.17)	662 (20.51)	49 (1.74)
Vocational Training	36 (2.61)	22 (1.91)	87 (2.70)	95 (3.38)
Marginally Employed	8 (0.58)	6 (0.52)	90 (2.79)	40 (1.42)
ST unemployment				
Yes	341 (19.02)	241 (13.98)	202 (4.96)	211 (5.16)
No	1452 (80.98)	1483 (86.02)	3872 (95.04)	3882 (94.84)
LT unemployment				
Yes	114 (6.36)	57 (3.31)	50 (1.23)	85 (2.08)
No	1679 (93.64)	1667 (96.69)	4024 (98.77)	4008 (97.92)
Unemployed 1993				
Yes	327 (19.87)	207 (5.49)	185 (12.04)	219 (5.76)
No	1319 (80.13)	3565 (94.51)	1351 (87.96)	3583 (94.24)
Unemployed 1994				
Yes	325 (20.41)	204 (5.65)	218 (14.45)	272 (7.57)
No	1267 (79.59)	3407 (94.35)	1291 (85.55)	3323 (92.43)
Unemployed 1995				
Yes	274 (17.96)	210 (14.22)	155 (4.54)	242 (7.06)
No	1252 (82.04)	1267 (85.78)	3257 (95.46)	3184 (92.94)
Unemployed 1996				
Yes	254 (17.39)	169 (12.57)	186 (5.70)	229 (7.13)
No	1207 (82.61)	1176 (87.43)	3078 (94.30)	2983 (92.87)

*ST unemployment:* registered unemployed in 1991 or 1992, *LT unemployment:* registered unemployed in 1991 and 1992; *N.B.* The data for the control variables (age, household income, partnership status, education, employment type) and social context comes from wave 1992

### Measures

All methods were performed in accordance with the relevant guidelines and regulations (Declaration of Helsinki). Self-rated health was measured using the item „How would you describe your current health?”. The 5-point scale – *1* (very good) to *5* (bad) – was inverted so that higher values reflected better self-rated health. This item was asked infrequently in the GSOEP from 1992 onwards; we used the data from 1992, 1994 and 1996 to reflect the time shortly after German reunification.

Life satisfaction was measured using the item „How satisfied are you with your life overall?“. An 11-point scale of *0* (completely dissatisfied) to *10* (completely satisfied) was used, so that higher values reflected greater life satisfaction. This item was asked annually, so data from the waves 1992 to 1996 was used.

Unemployment was measured by asking “Are you officially registered unemployed at the Federal Employment Agency?”, with *yes* or *no* as possible answers. For each year (1991–1996) this variable was recoded to *1* for *unemployed* and *0* as *not unemployed*. To reflect unemployment directly after reunification (1991 & 1992) two dummy-coded variables were computed: *short-term unemployment* (1 = unemployed in either years, 0 = not unemployed during either, 0 = unemployed in both) and *long-term unemployment* (1 = unemployed both years, 0 = not unemployed during either, 0 = unemployed in either years).

Older age [8] as well as lower income and education [3] are associated with worse health in unemployed individuals. Also, traditional gender roles in respect to employment have been shown to be of importance [40, 43]. Taking a similar approach to previous studies (e.g., [19]), the following were included as control variables in all analyses: age, household income, education and partnership status. Values from wave 1992 were used for the variables age (years), household income (Euros), gender (1 = female, 0 = male), partnership status (1 = in partnership, 0 = not in partnership), education (contrast 1: 1 = medium, 0 = low, 0 = high; contrast 2: 1 = high, 0 = low, 0 = medium) and social context (1 = East, 0 = West).

### Statistical analysis

**Fig. 1:**
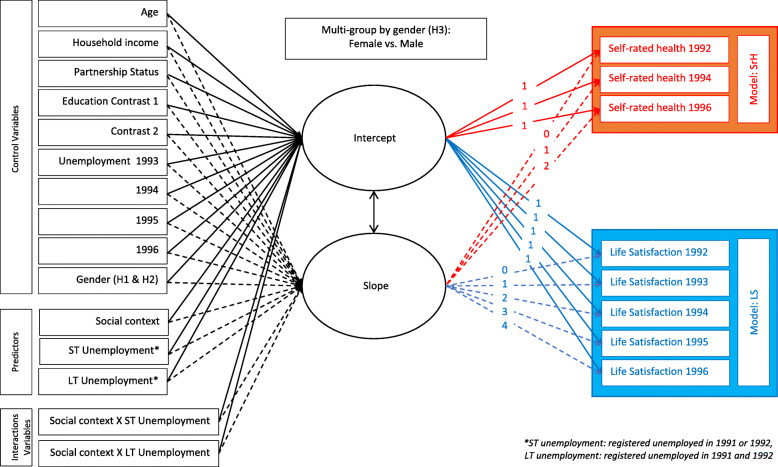
Statistical Model

To address the current research question latent growth modelling (LGM) was conducted with the Lavaan package [47] in R version 3.6.2 [48]. Growth analysis “attempt [s] to estimate between-person differences in within-person change” [49]. Recently LGM has been drawing on confirmatory factor analysis and structural equation modelling methods in a two-stage process. First, multiple measurement points of the outcome are fitted to latent factors of intercept (i.e. baseline value) and slope (i.e. growth). In the second stage, additional – e.g. inter-person – variables are entered as predictors of the longitudinal growth trajectories [49, 50]. See Fig. 1 for a visualization of the present statistical models. LGMs were computed separately for each health outcome (self-rated health and life satisfaction). The models were run with the whole sample to address *hypotheses 1 and 2* and then separately by gender to address *hypothesis 3*.

While growth modelling is very flexible, some basic requirements need to be met. First, similar to other statistical analyses, an adequate sample is required [49] – with a few thousand participants, this criterion was met in the present data.

Second, while there is no precise rule of thumb, the use of a minimum of three waves is generally advocated, with more data naturally offering more accurate models [49]. Again, this criterion was fulfilled, as the present study used data from 1992 to 1996 (i.e. three waves for self-rated health and five waves for life satisfaction). To account for the difference in number of time points between the outcome variables, we also ran adjusted models with life satisfaction from the waves 1992, 1994 and 1996 only. The pattern of associations did not change, only the effect sizes reduced from the three- to five-wave models. Also, in the three-wave latent growth models (LGMs), the interaction social context and long-term unemployment was significant in the male sample and this became non-significant in the five-wave LGMs. As Curran et al. [49] suggest that model accuracy increases with more data, the five-wave models are used for all analyses.

Similar to previous studies [51] we assumed a linear progression and therefore fixed the intercept values at 1.0 and the slope values at 0.0, 1.0, 2.0 and 0.0, 1.0, 2.0, 3.0, 4.0 for self-rated health and life satisfaction respectively. The final criterion was also fulfilled, namely the outcome measures being continuous and normally distributed [49].

To evaluate the models, Hu and Bentler’s [52] guidelines for model fit indices were used: Root Mean Squared Error of Approximation (RMSEA) < .06, Comparative Fit Index (CFI) > .95 and Standardized Root Mean Square Residual (SRMR) < .08. For model comparisons the Bayesian Information Criterion (BIC) and the Akaike Information Criterion (AIC) were used to rank order models, with the lowest BIC and AIC values indicating better models [49].

## Results

To gain an initial understanding of the predictor and outcome variables Pearson correlations were run separately by gender (see Additional file 1).

For all LGMs the fit indices showed a very good fit (see Table 2). The associations (effect size and significance) between control, predictor and outcome variables did not alter greatly when the interaction terms were entered into the models, therefore only the values of the models with interaction terms are reported.

**Table 2:** Results of latent growth models (LGM) by total, female, and male sample (standardized *β*-values)

		Self-rated health						Life satisfaction					
				female		male				female		male	
		I	S	I	S	I	S	I	S	I	S	I	S
Age		**−.48**	**.13**	**−.47**	*.15*	**−.50**	**.13**	*−.04*	−.01	*−.06*	−.00	−.01	−.03
Household income		**.08**	.00	**.09**	−.02	**.07**	.02	**.12**	−.05	**.12**	−.04	**.12**	−.06
Partnership status		−.02	*.08*	−.02	.11	−.01	.07	**−.05**	.03	*−.06*	.05	−.03	.01
Education													
Contrast 1		.02	.04	.01	.05	.03	.04	.01	.00	.00	−.01	.01	.01
Contrast 2		**−.08**	.00	**−.08**	.02	**−.08**	.01	−.00	−.02	.01	−.02	−.01	−.03
Unemployment													
1993		.01	−.01	.05	−.05	−.02	.01	**−.07**	**.08**	**−.10**	**.16**	−.05	.00
1994		−.03	.01	−.03	.01	−.03	.02	**−.10**	**.09**	**−.10**	.06	**−.11**	**.13**
1995		−.04	**.07**	*−.08*	.11	.00	.06	−.04	−.05	*−.06*	−.03	−.02	−.07
1996		*.04*	−.01	.00	−.07	**−.08**	.02	.00	**−.19**	.03	**−.18**	−.03	**−.20**
Gender		**.08**	−.05					−.02	.01				
Social context		**.10**	**−.16**	**.09**	−.16	**.10**	**−.16**	**−.31**	**.23**	**−.29**	**.20**	**−.33**	**.26**
ST Unemployment		−.05	−.01	−.10	.05	−.01	−.04	**−.08**	.07	**−.11**	.08	−.04	.06
LT Unemployment		**−.09**	.00	*−.12*	.03	−.06	−.02	**−.09**	.07	−.04	−.01	**−.12**	*.13*
*Social context* X ST *Unemployment*		.06	−.03	.10	−.11	.01	.02	−.03	.04	−.01	.04	−.04	.05
*Social context* X LT *Unemployment*		.06	.01	.08	−.00	.03	.02	.04	−.01	.01	.03	.04	−.02
I – S correlation	S mean	**−.24**	**−1.87**	−.17	**−2.38**	−.24	−1.92	**−.25**	**.79**	**−.24**	.59	**−.24**	**1.04**
CFI		1.00		1.00		1.00		1.00		1.00		1.00	
RMSEA	SRMR	.04	.01	.04	.01	.04	.01	.03	.01	.04	.02	.03	.01
AIC	BIC	46,839.72	47,099.27	24,393.39	24,614.89	22,445.57	22,665.95	119,610.20	119,882.93	61,416.62	61,650.01	58,199.01	58,431.12
model *X*^2^		*X*^2^(16) = **148.51**		*X*^2^(15) = **78.59**		*X*^2^(15) = **81.09**		*X*^2^(55) = **458.59**		*X*^2^(52) = **279.54**		*X*^2^(52) = **241.30**	
*N*		6838		3473		3365		6756		3435		3321	

Test significance: **bold:** ***p*** **< .001**, *italics: p < .01*, underlined: p < .05; *ST unemployment*: registered unemployed in 1991 or 1992; *LT unemployment*: registered unemployed in 1991 and 1992; *I*: Intercept; *S*: Slope; *RMSEA*: root mean squared error of approximation; *CFI*: comparative fit index; *SRMR*: Standardized Root Mean Square Residual; *BIS*: Bayesian Information Criterion; *AIC*: Akaike Information Criterion; Analyses were also run on a subsample, retaining full-time, part-time and not employed participants. As the effect sizes and directions did not change drastically, the broader sample was retained

Of the control variables, age was the strongest predictor of self-rated health, while household income was the strongest predictor of life satisfaction. Greater age, but lower household income were associated with a lower intercept and steeper decline of both self-rated health and life satisfaction. Being in a partnership significantly predicted a worse starting point of life satisfaction. The highest versus lowest education was associated with a lower intercept in self-rated health. There were multiple but no consistent associations between unemployment in 1993–1996 and the health outcomes.

### Short- versus long-term unemployment

**Fig. 2:**
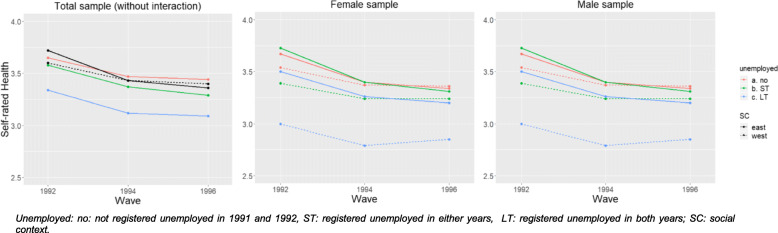
Descriptive trajectories of self-rated health (waves 1992, 1994, 1996)

**Fig. 3:**
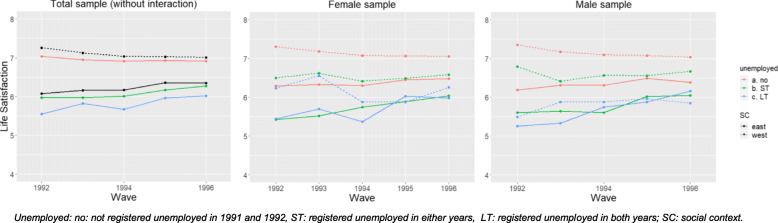
Descriptive trajectories of life satisfaction (waves 1992, 1993, 1994, 1995, 1996)

Figures 2 and 3 show the descriptive trajectories of self-rated health and life satisfaction respectively. The descriptive trajectories of self-rated health were worse for participants reporting long-term, compared to no or short-term unemployment after reunification. The results from the LGMs with the whole sample supported this pattern, as only long-term unemployment was significantly related to a lower starting point of self-rated health.

For life satisfaction, the descriptive trajectories had a lower starting point for short- and long-term compared to no unemployment. The LGMs supported this relationship, as both types of unemployment were significantly associated with a lower starting point of life satisfaction.

Thus, the effect of long- versus short-term unemployment on self-rated health was more negative, while the effects of short- and long-term unemployment were similar on life satisfaction.

### Social context

The descriptive trajectories of both health outcomes were generally lower in the West versus East German sample. The results from the LGMs with the whole sample confirmed a significant direct effect of social context on both aspects of health. East Germans showed a higher starting point of self-rated health and more negative trajectories, but a lower starting point of life satisfaction and more positive trajectories. Notably the effect was stronger with life satisfaction compared to self-rated health.

To address *H2* the interaction effect of social context and unemployment was inspected. West German men and women who were unemployed long-term after reunification had lower starting points of self-rated health and their trajectories did not catch up to the other groups. The LGM showed significant interaction effects of both types of unemployment in respect to the intercept of self-rated health, with reported unemployment being associated with a higher starting point in the East German sample.

The descriptive trajectories of life satisfaction showed better satisfaction in persons who reported no unemployment compared to short- and long-term unemployment. However, the interaction on life satisfaction did not reach statistical significance in the LGMs. Thus, *H2* was only supported in respect to self-rated health and not life satisfaction.

### Gender effects

Next, the interaction term – social context and unemployment after reunification – was examined in men and women separately. The descriptive trajectories of West German men and women who reported long-term unemployment showed a lower intercept of self-rated health. The LGMs revealed that the interaction of social context with unemployment was only significantly positively associated with the intercept of self-rated health in the female, but not the male sample. West German women reported the lowest starting point for self-rated health following unemployment. This was the case for both short- and long-term unemployment after reunification. The fit indices however only confirmed that the model with long- versus short-term unemployment was a better fit in the female than the male sample.

The descriptive trajectories of life satisfaction divided by gender did not reveal a clear interaction effect of social context and unemployment and the LGMs also did not show significant associations between the interaction and life satisfaction in either gender.

Thus, *H3* was only confirmed in relation to self-rated health and not life satisfaction.

## Discussion

We examined the interplay of gender, social context and job loss on long-term effects of health in a period and social context where drastic unemployment divided a population. The findings revealed the importance of social context in the impact of unemployment and that this effect was not consistent across different health outcomes. While we found social context to be the strongest predictor of both baseline and the development of health, the most notable finding was the significant interaction of social context and unemployment with baseline self-rated health in the female sample. Data following German reunification was analysed, because East Germans were confronted with a major change in their employment system and they collectively faced a dramatic increase in unemployment [27]. This was also seen in this sample, where East compared to West Germans were 3.2 times as likely to be unemployed in either 1991 or 1992 and 2.9 times as likely to be unemployed in both years.

Similar to previous findings [43, 44], the interactions between unemployment, gender and psychological distress were dependent on the social context. We expected East German women to find it more difficult to recover following unemployment, because they would assign greater importance to employment compared to their West German counterparts. Instead, we found that the West German, female sample showed lower baseline self-rated health following unemployment and did not recover to the level of the other groups. Around the time of the German reunification, the majority of West German women followed traditional gender roles and were either housewives or in part-time employment [39]. Perhaps we drew on a subset of West German women: by working and therefore breaking with traditional gender roles, these women would have been more modern and independent than the average West German woman. Indeed, in the present West German, female sample, those who were unemployed in both 1991 and 1992 were 5.9 times less likely to be in a partnership compared to their East German counterparts. Being married has a protective effect on the mental health of unemployed women [53] – not being able to depend on a partner’s income would make them more reliant on their own employment. Thus, many more factors may have been coupled with their unemployment and given this event an even greater significance than mere financial strain; for instance, the pressures of succeeding against the odds in a male dominated society and, as a result of their job loss, apparently ‘failing’. In line with the view that unemployment and health have a reciprocal relationship [30], West German women possibly exhibited worse health prior to their job loss due to surrounding factors. Having said that, this is beyond the scope of the present investigation and future research may want to consider such aspects in more detail.

In comparison, the East German, female sample reacted more similarly to the male samples from both social contexts. In former East Germany, gender equality in income and employment was common practice [37]. Traditional gender roles were not the norm [27] and women were able to easily combine being a mother, wife and worker [38]. It follows then that after the reunification, like their male counterparts, East German women would have desired and felt obligated to continue their employment.

The trend we found was that East Germans and West German men reported a gradual decline in their self-rated health. Our results confirmed that high regional unemployment not only impacts those directly affected, but also employed persons in those areas [3, 35].

### Short- versus long-term unemployment

The present results confirmed that long- compared to short-term unemployment had a stronger impact on self-rated health but no difference was found in relation to life satisfaction [3, 6, 16, 18]. Long-term unemployment has often been defined as six to 12 months or more [21, 22], whereas we defined short-term unemployment as being registered unemployed in one year and long-term unemployment as being registered unemployed in two consecutive years. The only association we found with short-term unemployment were significantly, lower baseline values of life satisfaction. As the other associations with the trajectories of health were found with long-term unemployment, it appears that the definition of long-term unemployment as more than one year is not sufficient. Similarly Milner et al. [23] found that health risks were not constant during a period of unemployment and Flatau et al. [7] found that compared to men, women’s mental health fluctuated more frequently within the first year of unemployment. As we did not measure unemployment length in smaller increments, their results may explain why the female sample showed a lower starting point on both health outcomes following short-term unemployment. Namely, that following short-term job loss the women adjusted to the experience of unemployment and therefore their satisfaction levels were not as affected by further unemployment.

Instead we found that men who experienced long-term unemployment reported lower initial life satisfaction and showed positive trajectories. The male sample appeared to adjust to short-term unemployment more easily, but then were more affected by long-term unemployment. Drawing on the set-point theory [11, 12], job loss was proposed to throw persons from their equilibrium of health, to then recover to their original, ‘set-point’. Our results in relation to life satisfaction support set-point theory and indicated a recovery in men following long-term unemployment.

On the other hand, in relation to self-rated health both men and women who faced long-term unemployment reported significantly lower baseline values and their trajectories did not recover to the values of their employed counterparts. Our findings are parallel to the findings of Clark et al. [6], who reported that unemployed persons did not return to their baseline values of well-being long after the end of their unemployment. In respect to self-rated health, we also found that long-term unemployment established a new, lower set-point and the former levels were not recovered. Thus, our findings indicate that men facing long-term unemployment were able to recover in terms of life satisfaction, but their self-rated health showed a longer term setback.

### Strengths, limitations and future directions

One strength of the current investigation is that – unlike previous unemployment research – two health constructs as well as two social contexts were examined separately. As our findings revealed different effects for self-rated health and life satisfaction as well as across social contexts this approach was indeed beneficial.

Another strength is that long-term unemployment was measured as being registered unemployed in two consecutive years, while generally it has been defined as only more than one year [22]. Due to the operationalization of the short- and long-term unemployment construct, it is possible that participants were not unemployed continuously for one or two years, respectively. However, our analyses revealed greater effects for long-term unemployment and only minimal associations with short-term unemployment and support the importance of extending the definition of long-term unemployment. Further, while the unemployment measure can clearly distinguish unemployment from other types of non-employment (e.g., retirement, maternity leave), it possesses ambiguity in terms of employment type (e.g., full-time, part-time, vocational training).

As the case numbers would have been too low to consider the numerous predictor variables, the current sample did not solely consist of individuals who were solely unemployed or reemployed between 1992 to 1996. Significant effects were found above and beyond controlling for unemployment in the following years. Thus, the current approach can in fact be seen as a strength, namely that this study considered a non-induced phenomenon of mass unemployment.

Further, the effect sizes of the direct and interaction effects of unemployment were relatively small. The small effects of negative life events are well known(e.g., [54]) and indeed that the magnitude of the impact drops quickly after one year [55]. In turn, the fact that we did find long-term effects highlights the significance that unemployment has on health.

## Conclusions

We analysed data from a particularly stark turning point in German history, where mass unemployment was widespread and divided the population. Certainly, this was not the last wave of such mass unemployment in Germany or other parts of the world and so the present findings provide guidance to similar circumstances. Social context was the strongest predictor of the development of health, yet the most notable finding was that West as opposed to East German women reported lower baseline self-rated health. At the time, this group would have been expected to be least affected by the economic crisis. Yet it appears that when this group – that was likely already burdened by numerous other stressors, breaking with traditional gender roles and fighting against the odds in a male dominated society – were confronted with unemployment they faced just one too many stressors. This highlights that during an economic crisis, policies makers and health providers should not only target those most directly affected economically but should also focus on populations who already carry a higher burden and may then simply be tipped over the edge.

## Supplementary Information

**Additional file 1.** AF1. Correlation matrix of predictor and outcome variables (female sample above, male sample below the axis).

## Data Availability

The datasets generated and/or analysed during the current study are available in the repository of the German Institute for Economic Research, https://www.diw.de/en/diw_02.c.222843.en/ forms.html.

## Abbreviations

GSOEP: German Socio-Economic Panel
LGM: Latent growth model
RMSEA: Root mean squared error of approximation
CFI: Comparative fit index
SRMR: Standardized Root Mean Square Residual
BIC: Bayesian Information Criterion
AIC: Akaike Information Criterion
