# Supplementary material for: The interplay of gender, social context, and long-term unemployment effects on subjective health trajectories
Source: BMC Public Health. 2021 Feb 4;21:290. doi: 10.1186/s12889-021-10324-8 (PMC7859896; doi:10.1186/s12889-021-10324-8)
Supplement: Supplementary file 1 — Additional file 1. AF1. Correlation matrix of predictor and outcome variables (female sample above, male sample below the axis). [file 12889_2021_10324_MOESM1_ESM.docx]

# Additional Files

AF1. Correlation matrix of predictor and outcome variables (female sample above, male sample below the axis).

|  | 1 | 2 | 3 |  | 4 | 5 | 6 |  | 7 | 8 | 9 | 10 | 11 |
| --- | --- | --- | --- | --- | --- | --- | --- | --- | --- | --- | --- | --- | --- |
|  | Social | Unemployment | |  | Self-rated health | | |  | Life satisfaction | | | | |
|  | context | ST | LT |  | 1992 | 1994 | 1996 |  | 1992 | 1993 | 1994 | 1995 | 1996 |
| 1 |  | **.21** | **.14** |  | **.05** | .01 | .00 |  | **-.31** | **-.26** | **-.24** | **-.19** | **-.17** |
| 2 | **.15** |  | **-.05** |  | .01 | -.01 | -.02 |  | **-.18** | **-.15** | **-.13** | **-.12** | **-.10** |
| 3 | *.03* | *-.04* |  |  | *-.05* | **-.05** | **-.05** |  | **-.12** | **-.09** | **-.12** | **-.07** | **-.07** |
| 4 | **.04** | **-.04** | **-.06** |  |  | **.54** | **.53** |  | **.31** | **.24** | **.25** | **.24** | **.26** |
| 5 | -.02 | *-.03* | **-.06** |  | **.57** |  | **.55** |  | **.26** | **.28** | **.37** | **.32** | **.31** |
| 6 | -.03 | **-.05** | **-.06** |  | **.54** | **.60** |  |  | **.25** | **.24** | **.28** | **.33** | **.41** |
| 7 | **-.33** | **-.13** | **-.12** |  | **.31** | **.29** | **.26** |  |  | **.56** | **.49** | **.47** | **.43** |
| 8 | **-.25** | **-.13** | **-.10** |  | **.26** | **.31** | **.27** |  | **.58** |  | **.57** | **.52** | **.47** |
| 9 | **-.21** | **-.11** | **-.08** |  | **.27** | **.42** | **.32** |  | **.52** | **.56** |  | **.57** | **.51** |
| 10 | **-.17** | **-.09** | **-.07** |  | **.28** | **.34** | **.35** |  | **.50** | **.55** | **.59** |  | **.57** |
| 11 | **-.17** | **-.08** | **-.07** |  | **.26** | **.33** | **.41** |  | **.44** | **.51** | **.53** | **.58** |  |
| Test significance: **bold: *p* <.001**, *italics:* *p < .01*, underlined: *p* < .05; ST unemployment: registered unemployed in 1991 or 1992, LT unemployment: registered unemployed in 1991 and 1992 | | | | | | | | | | | | | |
